# Absence of Thyroid Hormone Induced Delayed Dendritic Arborization in Mouse Primary Hippocampal Neurons Through Insufficient Expression of Brain-Derived Neurotrophic Factor

**DOI:** 10.3389/fendo.2021.629100

**Published:** 2021-02-23

**Authors:** Hiroyuki Yajima, Izuki Amano, Sumiyasu Ishii, Tetsushi Sadakata, Wataru Miyazaki, Yusuke Takatsuru, Noriyuki Koibuchi

**Affiliations:** ^1^Department of Integrative Physiology, Gunma University Graduate School of Medicine, Maebashi, Japan; ^2^Education and Research Support Center, Gunma University Graduate School of Medicine, Maebashi, Japan; ^3^Department of Bioscience and Laboratory Medicine, Hirosaki University Graduate School of Health Sciences, Hirosaki, Japan; ^4^Department of Medicine, Johmoh Hospital, Maebashi, Japan; ^5^Department of Nutrition and Health Sciences, Toyo University, Itakura, Japan

**Keywords:** hypothyroidism, brain-derived neurotrophic factor, Sholl analysis, dendrite growth, primary culture, hippocampus

## Abstract

Thyroid hormone (TH) plays important roles in the developing brain. TH deficiency in early life leads to severe developmental impairment in the hippocampus. However, the mechanisms of TH action in the developing hippocampus are still largely unknown. In this study, we generated 3,5,3’-tri-iodo-l-thyronine (T_3_)-free neuronal supplement, based on the composition of neuronal supplement 21 (NS21), to examine the effect of TH in the developing hippocampus using primary cultured neurons. Effects of TH on neurons were compared between cultures in this T_3_-free culture medium (-T_3_ group) and a medium in which T_3_ was added (+T_3_ group). Morphometric analysis and RT-qPCR were performed on 7, 10, and 14 days *in vitro* (DIV). On 10 DIV, a decreased dendrite arborization in -T_3_ group was observed. Such difference was not observed on 7 and 14 DIV. Brain-derived neurotrophic factor (*Bdnf*) mRNA levels also decreased significantly in -T_3_ group on 10 DIV. We then confirmed protein levels of phosphorylated neurotrophic tyrosine kinase type 2 (NTRK2, TRKB), which is a receptor for BDNF, on 10 DIV by immunocytochemistry and Western blot analysis. Phosphorylated NTRK2 levels significantly decreased in -T_3_ group compared to +T_3_ group on 10 DIV. Considering the role of BDNF on neurodevelopment, we examined its involvement by adding BDNF on 8 and 9 DIV. Addition of 10 ng/ml BDNF recovered the suppressed dendrite arborization induced by T_3_ deficiency on 10 DIV. We show that the lack of TH induces a developmental delay in primary hippocampal neurons, likely caused through a decreased *Bdnf* expression. Thus, BDNF may play a role in TH-regulated dendritogenesis.

## Introduction

Thyroid hormone (TH) plays important roles in general body growth, cardiovascular system coordination, skeletal muscle regulation, and central nervous system (CNS) development (1, 2). Especially, TH deficiency from the embryonic to neonatal period causes various developmental disorders, including in the brain, that in humans were originally called cretinism (3, 4). Perinatal TH deficiency induces brain dysfunction such as disrupted motor coordination, memory impairment, and decline in intelligence quotient (IQ) score (5, 6). Thus, the presence of TH during the perinatal period is essential for normal brain development.

The hippocampus is one of the brain regions strongly affected by perinatal TH deficiency (7, 8). As a part of the cerebral limbic system, the hippocampus is mainly responsible for memory and learning (9). Various symptoms from perinatal TH deficiency have been clarified in the hippocampus of animal models. Impairment of spatial memory caused by perinatal TH deficiency has been reported in the Morris water maze test and in the object location test (10–12). The mechanisms of these behavioral changes are considered to be induced by the reduction of synaptic transmission efficiency and neurotransmitter release in the hippocampus in TH-deficient mice (10) and rats (13–16). Morphologically, a decrease in the volume of the hippocampal CA1 and CA3 region, as well as a decrease in granule and pyramidal cell dendrite arborization, has been reported in the rat hippocampus (17–20).

Appropriate dendrite development represents a key element in generating neuronal networks required for normal brain function (21). TH deficiency leads to neurodevelopmental delay (22) and a decrease in dendrite arborization in various brain regions (19, 20, 23). In particular, decreased dendrite arborization of Purkinje cells and delayed maturation of granule cells have been reported in the rodent cerebellum (24, 25). The reduction of dendrite arborization of pyramidal cells and granular cells in the rat hippocampus has also been reported (19, 20). However, a limited number of studies have reported on the dendrite morphology in the hippocampus during the early developmental period with hypothyroid condition. In addition, the key molecules for induction of the decrease in dendrite arborization by TH deficiency have not yet been identified (19).

Here, we describe the action of 3,5,3’-tri-iodo-l-thyronine (T_3_), an active form of TH, on the growth of hippocampal neurons. Neurons in each brain region have their specific dendritic morphology, which may be mimicked in culture (26). It is relatively easy to study the morphology of the neuron and molecular mechanism in primary culture system. Therefore, we established a primary culture system of hippocampal neurons with or without T_3_ and analyzed the changes in their morphology and mRNA levels that may be involved TH-mediated brain development. We analyzed the dendrite morphology by Sholl analysis and the number of dendrite branches. We confirmed the mRNA levels of TH related genes, TH receptor genes, and synapse-related genes. TH related genes: neurotrophin 3 (*Ntf3*) and brain-derived neurotrophic factor (*Bdnf*) both of which are neurotrophic factor, neurogranin (*Nrgn*) which is calmodulin-binding protein, and *hairless* (*Hr*) which is lysine demethylase and nuclear receptor corepressor were reported to be regulated by TH (25, 27). TH receptor genes: TH receptor alpha (*Thra*) and TH receptor beta (*Thrb*) were selected because the action of TH is mostly through these receptors (28). Synapse-related genes: synapsin I (*Syn1*) and synaptophysin (*Syp*) both of which are pre-synapse related protein, and disks large membrane-associated guanylate kinase scaffold protein 4 (*Dlg4*) which is post-synapse related gene were selected to examine the effect of TH deficiency on the synapse. Cytoskeleton-associated gene: microtubule associated protein 2 (*Map2*) was selected because it is important molecule for dendrite elongation (29). We found that T_3_ is involved in dendrite development during the growth process in the primary cultured hippocampal neurons. Furthermore, the decrease in brain-derived neurotrophic factor (BDNF) induced by TH deficiency may play a major role in the reduction of dendrite arborization.

## Materials and Methods

The experiments conducted in accordance with the guidelines and protocols were approved by the Animal Care and Experimentation Committee of the Gunma University (authorization number: 19-041). In this study, all efforts were made to minimize the suffering of animals and number of animals used.

### Cell Culture

The formulation of the neuronal supplement 21 (NS21) defined supplement for serum-free neuronal culture media has previously been described in this study by Chen et al. (30). Based on the protocol in this paper, we generated a handmade neural supplement that does not contain T_3_ (**Table 1**) used for our cell culture system.

**Table 1:** Composition of the neural supplement.

	Cat. #	Final medium concentration	
		μg/ml	μM
Albumin, bovine	A4919, Sigma-Aldrich	2,500	37
Catalase	C40, Sigma-Aldrich	2.5	0.010
Glutathione	G6013, Sigma-Aldrich	1.0	3.2
Insulin	I1882, Sigma-Aldrich	4.0	0.6
Superoxidase dismutase	S5395, Sigma-Aldrich	2.5	0.077
Holo-Transferrin	616424, Miltenyi Biotec	5.0	0.062
l-Carnitine	C7518, Sigma-Aldrich	2.0	12
Ethanolamine	E9508, Sigma-Aldrich	1.0	16
d(+)-Galactose	G0625, Sigma-Aldrich	15	83
Putrescine	P5780, Sigma-Aldrich	16.1	183
Sodium selenite	S9133, Sigma-Aldrich	0.01435	0.083
Corticosterone	C2505, Sigma-Aldrich	0.02	0.058
Linoleic acid	L1012, Sigma-Aldrich	1.0	3.5
Linolenic acid	L2376, Sigma-Aldrich	1.0	3.5
Lipoic acid (thioctic acid)	T1395, Sigma-Aldrich	0.047	0.2
Progesterone	P8783, Sigma-Aldrich	0.0063	0.02
Retinol acetate	R7882, Sigma-Aldrich	0.1	0.2
Retinol, all trans (vitamin A)	95144, Sigma-Aldrich	0.1	0.3
d,l-alpha-Tocopherol (vitamin E)	95240, Sigma-Aldrich	1.0	2.3
d,l-alpha-Tocopherol acetate	T3001, Sigma-Aldrich	1.0	2.1

**Figure 1:**
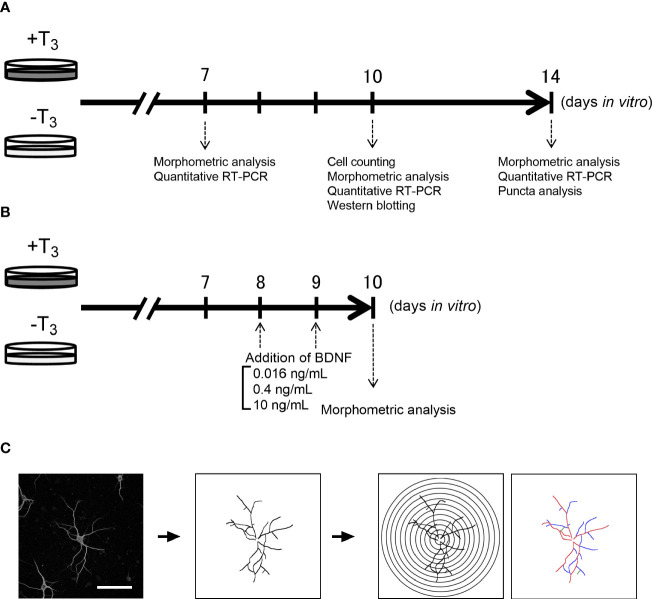
Schematic diagram of the experiment. Schedule of the overall experiment **(A)** and rescue experiment **(B)**. Schematic diagram of Sholl analysis and quantification of the number of dendrite branches (primary dendrite branch: red, secondary dendrite branch: blue, tertiary dendrite branch: green) **(C)**. Scale bar indicates 100 μm.

Pregnant C57BL/6J mice (gestation day 15/16) were purchased from SLC Japan (Hamamatsu, Shizuoka, Japan). The pregnant mice were decapitated under ketamine/xylazine anesthesthetic, and pups were taken out by cesarean section. After killing of the pups by hypothermia on ice, the hippocampus was removed (55 pregnant mice were used in total and 6–10 pups were taken from each pregnant mouse). Each culture contains cells of pups that were dissected out from the same dam. Brain tissues of pups from different dams were not mixed. Detailed methods for low-density culture of hippocampal neurons have previously been described (26, 31, 32). Briefly, the hippocampal tissue was digested for 10 min with shaking at 37°C using 15 U/ml of papain (Worthington Biochemical, Lakewood, NJ, USA) in phosphate buffered saline (PBS) containing 0.2 mg/ml L-cysteine, 0.2 mg/ml bovine serum albumin (BSA) (Thermo Fisher Scientific, Waltham, MA, USA), 5 mg/ml glucose, and 0.2 mg/ml DNase I (Sigma-Aldrich, St. Louis, MO, USA). Dissociated cells were placed at a density of 5,000–6,000 cells on 1 mg/ml poly-L-lysine (Sigma-Aldrich) coated round glass coverslips (12 mm in diameter) in a 24-well-plate. We placed one coverslip in each culture well. Cells were maintained in MACS Neuro Medium (Miltenyi Biotec, Bergisch Gladbach, Germany) containing 2% original neural supplement without T_3_ (**Table 1**), 1% 200 mM L-Glutamine (Thermo Fisher Scientific), and 1% 25 mM HEPES. Then, T_3_ dissolved in MACS Neuro Medium was added in some cultures at a final concentration of 3.0×10^−9^ M, the same concentration as for NS21 (+T_3_ group). The other group (-T_3_ group) received only vehicle. Cells were cultured in a 5% CO_2_ incubator at 37°C until 7, 10, and 14 DIV (**Figure 1A**). On 8 and 9 DIV, some of -T_3_ group received BDNF (PEPROTECH, Rocky Hill, NJ, USA) dissolved in 0.1% BSA containing Milli-Q water at a final concentration of 0.016, 0.4, and 10 ng/ml (**Figure 1B**). The concentration of 10 ng/ml BDNF was selected for this study, because it is in physiological range (33) and was an effective concentration for morphological analysis in previous studies (34). Concentration of 0.016 ng/mL BDNF was chosen for the lowest concentration and was identified by ELISA from the culture medium on 3 DIV (35). Concentration of 0.4 ng/ml BDNF was selected for the middle concentration between 10 to 0.016 ng/ml. Other cultures received vehicle of BDNF (0.1% BSA containing Milli-Q water). These cultured cells were maintained until 10 DIV.

### Immunocytochemistry

Hippocampal neurons were visualized by immunostaining. The primary hippocampal neurons on coverslips were fixed with 4% paraformaldehyde in PBS and were incubated with NH_4_Cl (50 mM) in PBS for 10 min. Then, cells were permeabilized with 0.1% Triton X-100 in PBS. After blocking with 5% fetal bovine serum in 0.3% Triton X-100, cells were incubated with primary antibodies overnight at 4°C. The following antibodies were used for 7, 10, and 14 DIV: Alexa Fluor 488 conjugated anti-MAP2 (Millipore Cat# MAB3418X, RRID: AB_571048; 1:1,000) and anti-synaptophysin (Cell Signaling Technology Cat# 5461P, RRID: AB_10838404; 1:1,000) antibody. The following antibodies were used for 10 DIV: anti-phosphorylated neurotrophic tyrosine kinase type 2 receptor (NTRK2) (Millipore Cat# ABN1381, RRID: AB_2721199, 1:500), anti-NTRK2 (BD Biosciences Cat# 610101, RRID: AB_397507, 1:1,000), anti-GFAP (Abcam Cat# ab7260, RRID: AB_305808, 1:1,000), and Alexa Fluor 488 conjugated anti-MAP2 antibody. The coverslips were rinsed with PBS and incubated with a secondary antibody conjugated with Alexa Fluor 596 Goat anti-rabbit IgG (Abcam Cat# ab150080, RRID: AB_2650602; 1:1,000) or with a secondary antibody conjugated with Alexa Fluor 596 Goat anti-rabbit IgG (Abcam Cat# ab150080, RRID: AB_2650602; 1:1,000) and Alexa Fluor 488 Goat anti-mouse IgG (Thermo Fisher Scientific Cat# A-11001, RRID: AB_2534069, 1:1,000) antibody for 1 h at room temperature. The coverslips were washed for 5 min in PBS, incubated for 30 min in 300 nM DAPI (Thermo Fisher Scientific Cat# D1306, RRID: AB_2629482) and mounted on glass slides with a Fluoromount/Plus (Diagnostic BioSystems, Pleasanton, CA, USA).

### Quantification of Cell Number

Total cell number, neuronal cell number, and astrocyte cell number were acquired on 10 DIV with IN Cell Analyzer 2200 (GE Healthcare, Chicago, IL, USA). Sixteen fields were randomly selected by the computer from each coverslip, and average number of 16 fields in one coverslip per one dam dissection was used for statistical analysis. The images were analyzed using IN Cell Developer Toolbox v1.9 (GE Healthcare). Total cell number was recognized based on DAPI staining. Total neuronal number was identified based on MAP2 and DAPI staining. Total astrocyte number was identified based on glial fibrillary acidic protein (GFAP) and DAPI staining. Co-localized areas of MAP2 and DAPI, or GFAP and DAPI were extracted using a command of Segmentation, Sieve, and Dilation.

### Cell Viability Assay

We conducted the 3-(4,5-dimethylthiazol-2-yl)-5-(3-carboxymethoxyphenyl)-2-(4-sulfophenyl)-2H-tetrazolium (MTS) assay (Promega, Madison, WI, USA) to confirm the cell viability on 10 DIV, as described in previous report (36). The succinic dehydrogenase found in the active mitochondria caused the conversion of MTS into aqueous soluble formation. Hippocampal neurons were seeded into 96-well plate (1,000–2,000 cells per well) and were maintained with or without T_3_ medium. On 8 and 9 DIV, BDNF were added to some of the -T_3_ group medium at a final concentration of 0.016, 0.4, and 10 ng/ml. The medium received MTS reagent and was incubated for 4 h in 5% CO_2_ at 37°C. The absorbance of each well was measured at 490 nm by using a micro-plate reader (Molecular Devices, San Jose, CA, USA). Relative values were calculated as percentages values of the +T_3_ group. Data were obtained from three independent dissections.

### Morphometric Analysis

#### Sholl Analysis

We carried out a Sholl analysis for quantitative analysis of dendrite morphology by ImageJ Fiji software v1.51 (37–39) (**Figure 1C**). Images of MAP2- and DAPI-positive neurons on 7, 10, and 14 DIV were taken with a confocal microscope LSM880 (Carl Zeiss, Oberkochen, Germany). Neurons were selected based on the criteria; low dendrite entanglement with other neuronal dendrites, or no aggregation with other neurons. To avoid the objective bias, we adopted single-blind method. We analyzed neurons matching our search criteria. The coverslip was scanned from left top area to right top then right bottom to left bottom. Morphometric analysis of neurons matching the criteria was also done with this direction and continued until the number of neurons reached to twenty per one coverslip from one dam dissection, and 4–5 coverslips were imaged in each treatment group. The image of each neuron was manually traced on computer. The transparent layer which overlaid on the image of the neuron was used for tracing of the neuron. Manually traced images of neuron drawn in a transparent layer were used for Sholl analysis. The 10–300 μm equidistant concentric circles were overlaid on the image of the neuron centered on the cell body. The intersections between dendrites and each concentric circle were measured.

#### Counting the Number of Dendrites

We counted the number of primary, secondary, and tertiary dendrites per neuron to quantify the dendrite branches (**Figure 1C**) (40). The dendrite branches were manually counted from the images acquired, as described above.

#### Quantitative Analysis of the Pre-Synaptic Puncta

Using immunocytochemistry for synaptophysin, a representative presynaptic protein (23), we counted the number of pre-synaptic puncta. The images stained with MAP2, synaptophysin, and DAPI on 14 DIV were captured with a confocal microscope LSM880. For quantification of pre-synaptic puncta, digital images (1,192 × 1,192 pixels) were acquired. Spine density was manually determined along secondary or tertiary dendritic segments using the ImageJ Fiji software. The images of pre-synapse staining were subjected to a user-defined intensity threshold to select puncta. The threshold was not changed during all experiment. The pre-synaptic puncta were measured co-localized to MAP2 staining.

### Quantitative RT-PCR

Total RNA was extracted from primary hippocampal neurons on 7, 10, and 14 DIV using QIAzol Lysis reagent (QIAGEN, Hilden, Germany). We harvested cell from three coverslips per one dam dissection, combined, and extracted total RNA. cDNA was synthesized from 250 ng of total RNA using ReverTra Ace qPCR RT Master Mix with gDNA Remover (TOYOBO, Osaka, Japan). Total RNA with 260/280 absorbance ratio higher than 1.8 was used. Specific primer sets were used for the following TH regulated genes: *Ntf3*, *Nrgn*, *Bdnf* (exon IX), *Hr*; TH receptor genes: *Thra*, *Thrb*; synapse-related genes: *Syn1*, *Syp*, *Dlg4*, cytoskeleton-associated gene; *Map2*. Primer sequences are described in **Table 2**. The quantitative RT-PCR protocol for all mRNAs was as follows: 95°C for 60 s; followed by 40 cycles of amplification at 95°C for 15 s; and 60°C for 45 s. The data were analyzed using the delta-delta Ct method. Levels of measured mRNAs were normalized by those of glyceraldehyde 3-phosphate dehydrogenase (*Gapdh*) used as internal control. The amplification efficiency of each primer set was 90 to 105%.

**Table 2:** Primer sequences used for RT-qPCR.

Neurotrophin 3 (*Ntf3*)	Forward	tgccacgatcttacaggtga
	Reverse	agtcttccggcaaactcctt
Neurogranin (*Nrgn*)	Forward	gccagacgacgatattcttgac
	Reverse	tatcttcttcctcgccatgtgg
Brain-derived neurotrophic factor (*Bdnf*)	Forward	atccaaaggccaactgaagc
	Reverse	attgggtagttcggcattgc
Hairless (*Hr*)	Forward	ttggcccttgtaggaaatgc
	Reverse	tttcagcttggtgtgatggc
Thyroid hormone receptor alpha (*Thra*)	Forward	cgcttcaagaagtgcattgc
	Reverse	tcaatcagcttgcgtttggc
Thyroid hormone receptor beta (*Thrb*)	Forward	acaagcacccatcgtgaatg
	Reverse	tggcagctcacaaaacatgg
Synapsin I (*Syn1*)	Forward	tgtgcgtgtccagaagattg
	Reverse	acatggcaatctgctcaagc
Synaptophysin (*Syp*)	Forward	tttgccatcttcgcctttgc
	Reverse	gggtgcatcaaagtacacttgg
Disks large MAGUK scaffold protein 4 (*Dlg4*)	Forward	ggtcaacgacagcatcctg
	Reverse	atgacgtagaggcgaacgatg
Microtubule associated protein 2 (*Map2*)	Forward	atgaaggaaaggcaccacac
	Reverse	tggaaatccattggcgttgc
Glyceraldehyde 3-phosphate dehydrogenase (*Gapdh*)	Forward	tgcgacttcaacagcaactc
	Reverse	atgtaggccatgaggtccac

### Western Blot Analysis

The expression levels of proteins were determined by Western blot analysis, as described previously (41). Briefly, total proteins were extracted from primary cultured hippocampal neurons in a lysis buffer containing 20 mM Tris-HCl (pH 8.0), 137 mM NaCl, 10% Glycerol, 1% Nonidet P40, and proteinase inhibitors. We harvested cells from 8 coverslips per one dam dissection, combined, and extracted protein. Protein concentration was measured by BCA Protein assay kit (Thermo Fisher Scientific) and 30 µg of total protein was used. Total protein was separated by sodium dodecyl sulfate-polyacrylamide gel electrophoresis and transferred to the nitrocellulose membrane. The membrane was blocked with 5% BSA in Tris Buffered Saline with 1% Tween 20 for 1 h at 4°C. Immunoblotting was performed using specific antibodies. Phosphorylated NTRK2 was detected using a rabbit polyclonal antibody against phosphorylated NTRK2 (Millipore Cat# ABN1381, RRID : AB_2721199, 1:500). NTRK2 was detected using a mouse monoclonal antibody NTRK2 (BD Biosciences Cat# 610101, RRID : AB_397507, 1:1,000). β-actin was detected using rabbit polyclonal antibody against β-actin (Cell Signaling Technology Cat# 4967, RRID : AB_330288, 1:2,000). The primary and secondary antibodies were stripped using Restore stripping buffer (Thermo Fisher Scientific). NTRK2 was used for normalization.

### Statistical Analysis

After the morphometric analysis, the average value/neuron in each coverslip was used as a representative value. Each group consists of 4–5 coverslips obtained from different dam dissection. The average value of each group was calculated using this representative value and the statistical analysis was also performed using this representative value. For quantitative RT-PCR and Western blot analysis, as stated above, each sample was obtained from different dam dissection, indicating equal weighting of the value. Results are presented as mean ± standard error of the mean (SEM). GraphPad Prism v8.0 for Windows (GraphPad Software, San Diego, CA, USA) was used for statistical analysis. Statistical comparisons were performed by Student’s two-tailed *t*-test or by one- or two-way analysis of variance (ANOVA), followed by the Tukey’s test for *post hoc* analysis. Differences were considered significant at *p*-values <0.05.

## Results

### Absence of T_3_ Did Not Affect Cell Viability

**Figure 2:**
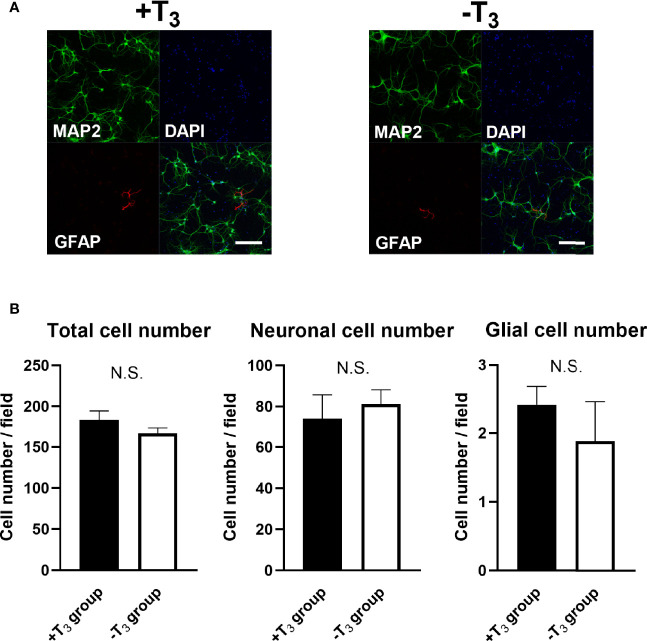
Absence of T_3_ did not change cell number on 10 DIV. **(A)** Representative images of the microtubule associated protein 2 (MAP2) (green), Glial fibrillary acidic protein (GFAP) (red), and DAPI (blue) immunoreactivities are shown. Scale bar indicates 200 μm. **(B)** Total cell number, Neuronal cell number, and astrocyte cell number were measured in +T_3_ and -T_3_ group using IN Cell Analyzer 2200. There were no significant differences in total cell number, neuronal cell number, and astrocyte cell number among groups on 10 DIV. Average number of 16 fields in one coverslip were used for statistical analysis. Data were obtained from four coverslips came from different dissections. (+T_3_ group, n = 4; -T_3_ group, n = 4). Graph shows means ± SEM.

To examine the cell viability, total cell number, neuronal cell number, and astrocyte cell number were assessed on 10 DIV using IN Cell Analyzer 2200. An average number of cells in 16 fields per one coverslip from one dam dissection was used for statistical analysis. Data were obtained from four coverslips from different dissection. There was no difference in total cell number and neuronal cell number between +T_3_ and -T_3_ group (**Figures 2A, B**). There are mainly three types of glial cell in the brain: astrocyte, oligodendrocyte, and microglia. We verified only the astrocyte number in the glial cells, because Xie et al. reported that oligodendrocyte and microglia could not survive in the neurobasal medium plus B27 supplement which is the origin of the NS21 (42). We examined the total astrocyte number using specific marker for astrocyte (anti-GFAP antibody) on 10 DIV. There was no difference in astrocyte cell number between +T_3_ and -T_3_ group on 10 DIV (**Figures 2A, B**). Cell viability was also investigated by MTS assay on 10 DIV. There was no difference among group (**Supplemental Figure 1**). These results indicate that absence of T_3_ did not induce hippocampal cell death.

### T_3_-Free Hippocampal Neurons Showed Growth Retardation

**Figure 3:**
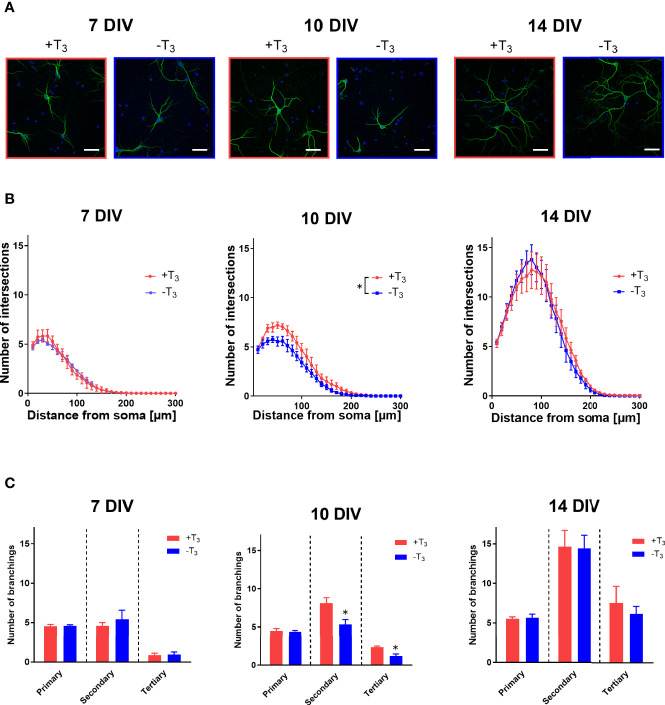
Hippocampal neurons cultured in T_3_-free medium showed growth retardation on 10 DIV. **(A)** Representative images of primary hippocampal neurons that were immunostained with a microtubule associated protein 2 (MAP2) antibody, from +T_3_ and -T_3_ group are shown. Scale bar indicates 50 μm. **(B)** The number of intersections of dendrite at each concentric circle, studied by Sholl analysis. Sholl results indicate that -T_3_ group shows less intersections on 10 DIV compared with +T_3_ group (*p < 0.05, two-way ANOVA). Data were obtained from 20 neurons per one coverslip. The average number of intersections were calculated and used as a representative number of each coverslip. Using this number, the average number of each group was calculated, and the statistical analysis was performed (7DIV: +T_3_ group, n = 4 coverslips; -T_3_ group, n = 4 coverslips; 10 DIV: +T_3_ group, n = 4 coverslips; -T_3_ group, n = 4 coverslips; 14 DIV: +T_3_ group, n = 5 coverslips; -T_3_ group, n = 4 coverslips). **(C)** In -T_3_ group, the number of secondary and tertiary dendrites significantly decreased on 10 DIV compared with +T_3_ group (*p < 0.05, *t*-test). Data analysis was performed as stated above (7DIV: +T_3_ group, n = 4; -T_3_ group, n = 4; 10 DIV: +T_3_ group, n = 4; -T_3_ group, n = 4; 14 DIV: +T_3_ group, n = 5; -T_3_ group, n = 4). Graphs in B, C show means ± standard error of the mean (SEM).

To quantify the dendrite morphology of the +T_3_ and -T_3_ groups, we performed a Sholl analysis, which is a method to determine dendrite arborization and extension (39) (**Figure 1C**). The intersections with dendrites and concentric circles increased along with growth between 7 and 14 DIV (**Figures 3A, B**). Representative data from each coverslip was obtained by counting 20 neurons. The average value of each group was calculated using the average value of each coverslip as a representative value of each coverslip. The statistical analysis was also performed using this representative values (see Materials and Methods). While the number of intersections was not different between +T_3_ and -T_3_ groups on 7 DIV, it decreased significantly on 10 DIV in -T_3_ group compared with +T_3_ group [*F*_(1, 176)_ = 44.88, *p* < 0.0001, ANOVA]. However, the difference was not observed on 14 DIV.

We next counted the number of primary, secondary, and tertiary dendrites to evaluate the effects of T_3_ on dendrite branching (**Figure 3C**). Data were obtained from 20 neurons and the data comparison and statistical analysis was performed as stated above. On 7 DIV, the average number of primary, secondary, and tertiary dendrites per neuron was similar in both groups. However, on 10 DIV, the average number secondary and tertiary dendrites decreased significantly in -T_3_ group [secondary: *t*_(7)_ = 2.87, *p* = 0.0241; tertiary: *t*_(7)_ = 3.52, *p* = 0.0098, *t*-test]. This difference was not observed on 14 DIV. These results indicated that the T_3_ exerts an effect on the morphology of primary hippocampal neurons only during a limited time window.

### Number of Pre-Synaptic Puncta Did Not Change by T_3_ Treatment

**Figure 4:**
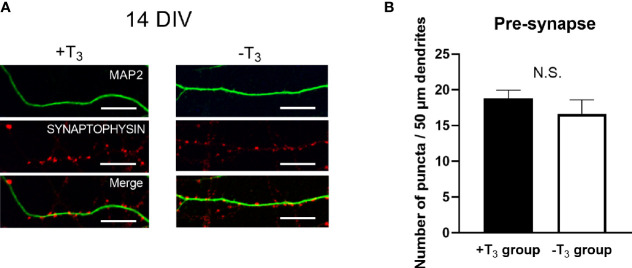
The number of synaptophysin-positive puncta on dendrites did not change in +T_3_ and -T_3_ group. **(A)** Representative images of the synaptophysin (red) on microtubule associated protein 2 (MAP2) (green) are shown. Scale bar indicates 10 μm. **(B)** The number of puncta in 50 µm dendrite. The number of dendritic puncta were not different between +T_3_ and -T_3_ groups. Twenty neurons were analyzed per one coverslip came from independent dissection. Then the data analysis was performed as indicated in the materials and methods section and in the legend of **Figure 3** (+T_3_ group, n = 4; -T_3_ group, n = 4). Graph in B shows means ± SEM.

We then counted the number of synaptic puncta to examine the change of pre-synapses by T_3_ (43, 44). Representative images of each group stained with synaptophysin (red) and MAP2 (green) are shown in **Figure 4A**. The number of synaptophysin-positive puncta on the 50 μm dendrite is shown in **Figure 4B**. Data were obtained from four coverslips came from different dissections; twenty neurons were analyzed per one coverslip. The average number of quantifications in each coverslip was used for statistical analysis as indicated above. The distribution of synapses was similar between +T_3_ and -T_3_ groups.

### mRNA Levels of Primary Hippocampal Neurons

**Figure 5:**
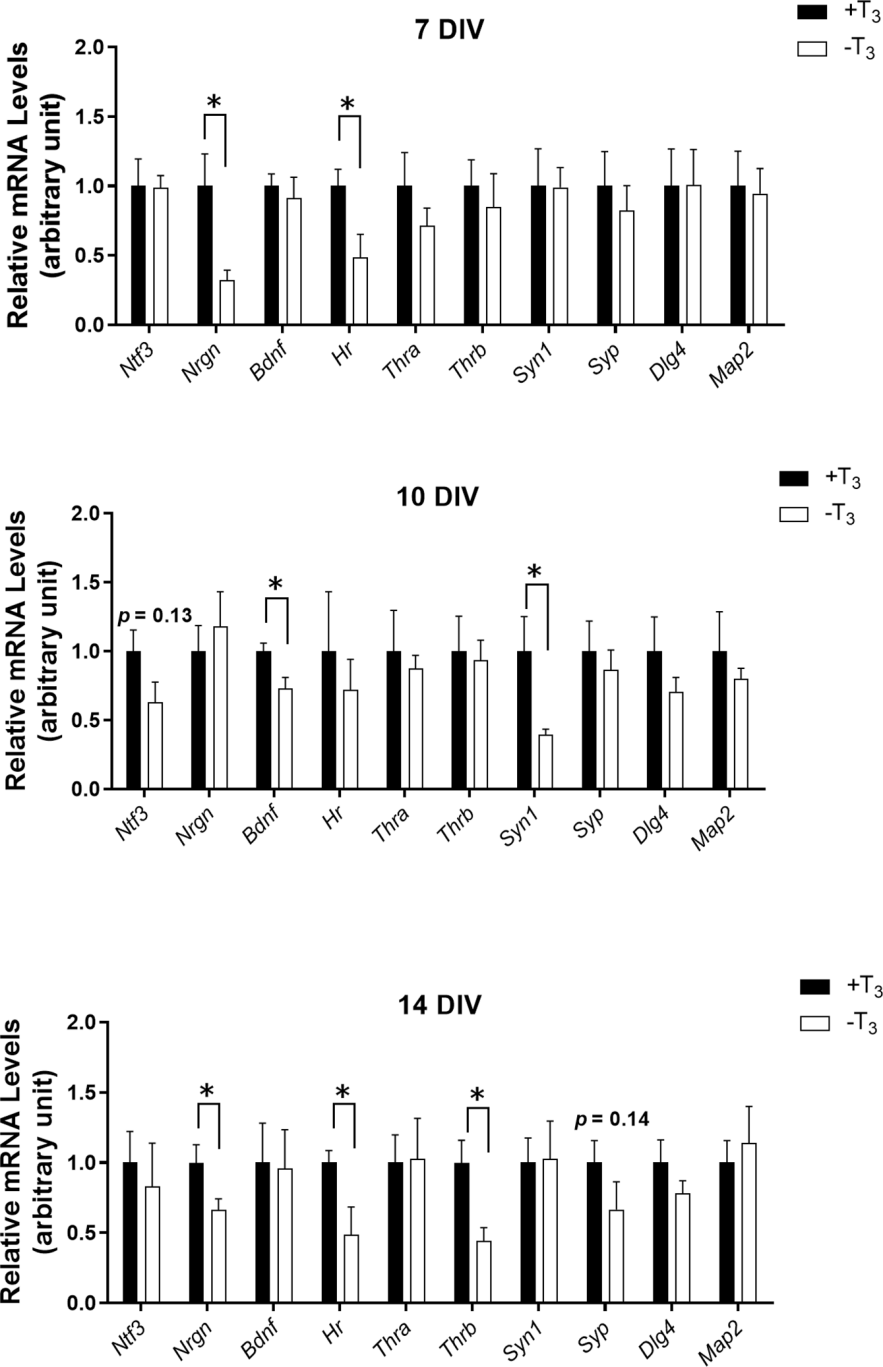
mRNA levels of TH-related genes and synaptic proteins of +T_3_ or -T_3_ group on 7, 10, and 14 DIV. The mRNA levels were normalized to glyceraldehyde 3-phosphate dehydrogenase (*Gapdh*) mRNA levels. We harvested cells from three coverslips per one dam dissection, combined all cells, extracted total RNA, and used for quantitative RT-PCR (7DIV: +T_3_ group, n = 5; -T_3_ group, n = 5; 10 DIV: +T_3_ group, n = 4–5; -T_3_ group, n = 5; 14 DIV: +T_3_ group, n = 5–6; -T_3_ group, n = 4–6). Asterisks indicate a statistical difference between +T_3_ and -T_3_ groups (*p < 0.05, *t*-test). Graph shows means ± SEM.

We performed quantitative RT-PCR to investigate changes in mRNA levels due to TH (**Figure 5**). We harvested cells from three coverslips per one dam dissection, combined all cells, and extracted total RNA. On 7 DIV, *Nrgn* and *Hr* mRNA levels decreased significantly in -T_3_ group compared with +T_3_ group [*Nrgn*: *t*_(8)_ = 2.81, *p* = 0.0228; *Hr*: *t*_(8)_ = 2.51, *p* = 0.0364, *t*-test]. On 10 DIV, when the abnormal dendrite arborization was observed, *Bdnf* and *Syn1* mRNA levels decreased significantly in -T_3_ group [*Bdnf*: *t*_(7)_ = 2.56, *p* = 0.0378, *t*-test; *Syn1*: *t*_(7)_ = 2.67, *p* = 0.0319, *t*-test]. At this period, despite growth retardation observed in -T_3_ group, the mRNA levels of cytoskeletal-related protein did not change. On 14 DIV, *Nrgn*, *Hr*, and *Thrb* mRNA levels decreased significantly in -T_3_ group [*Nrgn*: *t*_(10)_ = 2.23, *p* = 0.0495; *Hr*: *t*_(7)_ = 2.58, *p* = 0.0366; *Thrb*: *t*_(7)_ = 2.79, *p* = 0.0268, *t*-test].

Decreased dendrite arborization was observed on 10 DIV. However, this difference was not observed on 14 DIV and no significant changes were observed in the number of presynaptic puncta on 14 DIV. Quantitative RT-PCR results indicated that *Bdnf* mRNA levels significantly decreased on 10 DIV with decreased dendrite arborization, although TH receptor (TR) (*Thra* and *Thrb*) mRNA levels did not change on 10 DIV. Thus, the decreased dendrite arborization is likely caused by a mechanism mediated by TH.

### BDNF Rescued the Growth Retardation of T_3_-Free Hippocampal Neurons

**Figure 6:**
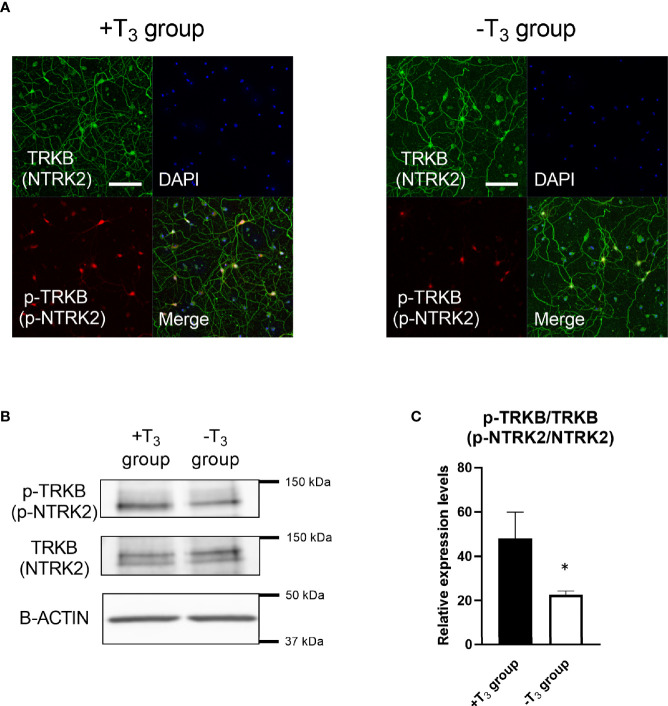
Phosphorylated tyrosine kinase receptor B (p-TRKB, p-NTRK2) levels of -T_3_ group is decreased compared with +T_3_ group. **(A)** Representative images of the TRKB (NTRK2) (green), and p-TRKB (red) immunoreactivity and DAPI (blue) staining are shown. Scale bar indicates 50 μm. **(B)** Representative result of Western blot for p-TRKB, TRKB, and β-actin. **(C)** The change in the ratio of p-TRKB/TRKB levels in Western blot. We harvested cell from 8 coverslips per one dam dissection, combined, extracted protein, and used for Western blot analysis. The p-TRKB levels decreased significantly in -T_3_ group (n = 8) compared with +T_3_ group (n = 5) (*p < 0.05, *t*-test). Data were obtained from five–eight independent dissection (+T_3_ group, n = 5; -T_3_ group, n = 8). TRKB were used for normalization. Graph shows mean ± SEM.

Based on the simultaneous decrease of *Bdnf* mRNA levels and abnormal dendrite arborization on 10 DIV, we examined whether BDNF is involved in this process. We confirmed the decrease in BDNF signaling in -T_3_ group on 10 DIV by checking the phosphorylation levels of NTRK2, which is a receptor for BDNF (**Figures 6A, B**). The phosphorylation of NTRK2 is triggered by binding with BDNF leading to the activation of downstream signaling pathways (45, 46). Thus, the phosphorylation levels of NTRK2 can be an indication of the signal intensity of BDNF. We conducted immunocytochemistry and Western blot analysis to verify the phosphorylated NTRK2 levels (**Figures 6A, B**). We harvested cells from eight coverslips per one dam dissection, combined, and extracted protein. In the immunocytochemistry study, -T_3_ group tended to show lower signal of phosphorylated NTRK2 compared with +T_3_ group (**Figure 6A**). In the Western blot study (**Figures 6B, C**), the phosphorylated NTRK2 levels of -T_3_ group significantly lowered compared with +T_3_ group [*t*_(11)_ = 2.69, *p* = 0.0211; *t*-test] (**Figure 6C**). These results indicate that BDNF involved in the dendrite arborization on 10 DIV.

**Figure 7:**
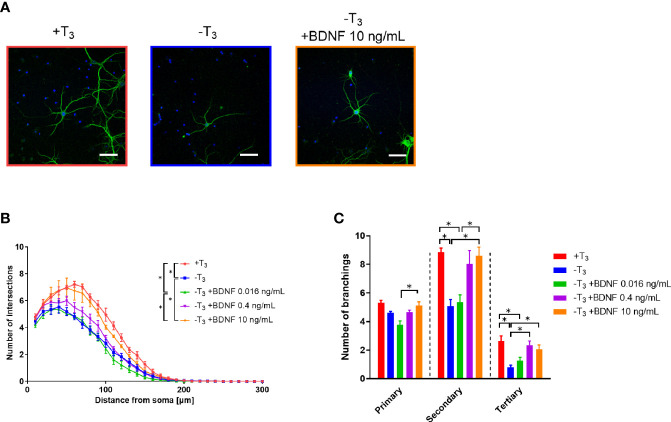
Addition of brain-derived neurotrophic factor (BDNF) rescued the decrease in dendrite arborization in -T_3_ group. **(A)** Representative images of MAP2 immunoreactivity showing the dendrite morphology of +T_3_, -T_3_, and the 10 ng/ml BDNF-supplemented group. Scale bar indicates 50 μm. **(B)** The number of intersections of dendrite at each concentric circle, studied by Sholl analysis. Sholl analysis indicates that +T_3_ and the highest BDNF concentration group are significantly different from -T_3_ group and the lowest BDNF concentration group (*p < 0.05, Tukey’s test). Data were obtained from 20 neurons per one coverslip came from independent dissection. Then the data analysis was performed as indicated in the materials and methods section and in the legend of **Figure 3** (+T_3_ group, n = 4; -T_3_ group, n = 4; -T_3_ +BDNF 0.016 ng/ml group, n = 4; -T_3_ +BDNF 0.4 ng/ml group, n = 4; -T_3_ +BDNF 10 ng/ml group, n = 4). **(C)** The addition of 10 ng/ml BDNF normalized the number of intersections similar to the +T_3_ group. Data were obtained from 20 neurons per one coverslip came from independent dissection. Then the data analysis was performed as indicated in the materials and methods section and in the legend of **Figure 3** (+T_3_ group, n = 4; -T_3_ group, n = 4; -T_3_ +BDNF 0.016 ng/ml group, n = 4; -T_3_ +BDNF 0.4 ng/ml group, n = 4; -T_3_ +BDNF 10 ng/ml group, n = 4). Graphs in B, C show means ± SEM.

We also examined whether BDNF addition ameliorates decrease in dendrite arborization on 10 DIV in -T_3_ group. BDNF at concentration of 0.016, 0.4, and 10 ng/ml was added to -T_3_ group culture on 8 and 9 DIV, and the dendrite arborization was measured by Sholl analysis on 10 DIV. The number of intersections increased by BDNF in a concentration-dependent manner (**Figures 7A, B**). The number of intersections was significantly different between +T_3_ group and -T_3_ group [*F*_(4, 449)_ = 86.91, *p* < 0.0001, two-way ANOVA, *p* < 0.0001, Tukey’s *post hoc* test], and between +T_3_ group and the lowest BDNF concentration (0.016 ng/ml) group [*F*_(4, 449)_ = 86.91, *p* < 0.0001, two-way ANOVA, *p* < 0.0001, Tukey’s *post hoc* test]. A significant difference in the number of intersections was observed between the group of highest BDNF concentration (10 ng/ml) and -T_3_ group [*F*_(4, 449)_ = 86.91, *p* < 0.0001, two-way ANOVA, *p* < 0.0001, Tukey’s *post hoc* test], and between the group with highest BDNF concentration and group with lowest BDNF concentration [*F*_(4, 449)_ = 86.91, *p* < 0.0001, two-way ANOVA, *p* < 0.0001, Tukey’s *post hoc* test]. There was no difference between +T_3_ group and the group with highest BDNF concentration.

We also counted the number of primary, secondary, and tertiary dendrites of +T_3_, -T_3_, and BDNF-supplemented groups (**Figure 7C**), respectively. The number of primary dendrites did not differ between the +T_3_ and -T_3_ group. The number of secondary dendrites as well as the -T_3_ group was significantly different between the +T_3_ group [*F*_(4, 15)_ = 9.225, *p* < 0.0006, one-way ANOVA, *p* = 0.0037, Tukey’s *post hoc* test] and the highest BDNF concentration (*p* = 0.0063, Tukey’s *post hoc* test).

Sholl analysis indicated that the decrease in dendrite arborization on 10 DIV was recovered by 10 ng/ml of BDNF treatment on 10 DIV. A concentration dependent increase of BDNF was observed in the number of secondary branches.

## Discussion

Herein, we investigated the effect of TH in the developmental process of hippocampal neurons in primary culture with or without T_3_. We analyzed the changes in dendritogenesis, synaptogenesis, mRNA levels, and protein levels. The decrease in dendrite arborization was observed only on 10 DIV (**Figure 3**). After this period, dendrite arborization caught up even without T_3_ and was at a level similar to +T_3_ group on 14 DIV. Because *Bdnf* mRNA levels also decreased in -T_3_ group on 10 DIV (**Figure 5**), we verified phosphorylated NTRK2 levels on 10 DIV to confirm the decrease in BDNF signaling in -T_3_ group. Phosphorylated NTRK2 levels significantly decreased in -T_3_ group on 10 DIV (**Figure 6**). We then attempted a rescue experiment by adding 10 ng/ml of BDNF to -T_3_ group (**Figure 7**). BDNF treatment rescued the phenotype of disrupted dendrite growth due to the absence of T_3_. indicating that BDNF is involved in TH-mediated dendrite arborization.

### Change of Gene Transcription Due to Thyroid Hormone Deficiency

TH is involved in the regulation of various genes expression (25, 47–49). Some of these genes are expressed in the brain and their expression is altered in perinatal hypothyroidism. The expression of the following neurotrophic factors; nerve growth factor (*Ngf*), *Ntf3*, and *Bdnf* has been reported to be altered by perinatal hypothyroidism. In a rat model of perinatal hypothyroidism, *Ngf* mRNA levels are decreased in the hippocampal dentate gyrus and CA1 region (50), and *Ntf3* mRNA levels are decreased in the dentate gyrus (50). Furthermore, *Bdnf* mRNA levels decrease on postnatal days (P) 15 and 30 in the rat perinatal hippocampus with hypothyroidism, whereas they return to normal on P60 (51). In the present study, T_3_ treatment did not affect *Ntf3* mRNA levels. On the other hand, *Bdnf* mRNA levels decreased in -T_3_ group specifically on 10 DIV (**Figure 5**). Moreover, our results are comparable to those in the study by Sui and Li et al., showing that the *Bdnf* mRNA levels in the rat hippocampus are normalized over time regardless of TH status. On the other hand, mRNA levels of TR, *Thra*, and *Thrb* were not altered by TH status (**Figure 5**). The general mechanism of transcriptional regulation of TR implies a TH-dependent activated transcription factor: when TH binds to TR, a co-activator protein complex is recruited by TR and target genes are transcribed (52). Therefore, the decrease in *Bdnf* mRNA levels in -T_3_ group is due to the lack of TH action, not to the difference in TR expression levels. Moreover, *Map2*, which is binding protein to the microtubules and is important component of dendrite elongation and stabilize microtubules (29), mRNA levels were similar in both groups, indicating that the change in dendrite development is not due to the change of *Map2* levels.

### Relationship Between Thyroid Hormone and Dendrite Arborization

BDNF belongs to the neurotrophin family, which plays important roles in neuronal survival and development (32, 53, 54). The action of BDNF on neuronal development has been well explored using knockout animals. Morphometric alterations such as reduction of dendrite arborization and extension have been reported in *Bdnf* gene conditional knockout mice (32, 55). Sui et al. demonstrated that perinatal hypothyroidism cause decreased BDNF transcription levels and expression levels during P1 to 30 in the hippocampus (51). Rami et al. revealed that decreased dendritic arborization occurred in the perinatal hypothyroid rat in the hippocampal granule and pyramidal cell on P6 and 10 (20). Thus, *Bdnf* is suggested to be involved in the TH-mediated development of hippocampal neurons. In this study, the reduction of *Bdnf* mRNA level observed on 10 DIV concomitant to the suppression of dendrite arborization in -T_3_ group. *Bdnf* gene is transcribed from multiple promoters (56). Based on a previous study showing that BDNF increased the number of primary dendrites in primary cultured hippocampal neurons (57), we also counted the number of dendritic branches. Although we could not clearly see the effect of BDNF on the development of primary dendrite branches in -T_3_ group, the deficient growth of secondary and tertiary branches was rescued by BDNF (**Figure 7**). Taken together, our study demonstrated that BDNF is involved in TH-mediated elongation and branching of dendrites in the hippocampus. However, the mechanism of BDNF action remains to be fully understood. Receptors for BDNF, such as NTRK2, are expressed in hippocampal neurons (58). Whether BDNF activates an intracellular signaling pathway through NTRK2 binding is yet to be demonstrated. Moreover, downstream targets inducing dendritogenesis remain to be identified and warrant further studies.

### Action of Thyroid Hormone on Synaptogenesis and Function

In the present study, we found that the decreased dendrite arborization occurred on 10 DIV and this alternation returned to normal on 14 DIV. This phenomenon is considered to be a “catch-up growth”. In addition to TH and BDNF, the dendritic growth is regulated by several other molecules (59). Although the mechanisms causing the catch-up growth cannot be clarified, such additional molecules may contribute to the “catch-up growth”. These results of the present study are analogous to the previous report of temporal morphological alternation in the cerebellum in thyroid hormone-deficient mice (24). This report indicated that despite morphological alternation return to normal, the impairment of synaptic function is remaining (24). TH is involved in synaptic formation and neuron functions (23, 60); for instance, the reduction of the number of synapses and the decrease in paired-pulse ratio, which is the indicator of calcium-dependent neurotransmitter release probability related to short-term synaptic plasticity (61), have been reported (15, 23, 24). Using synaptophysin immunocytochemistry (**Figure 4**), we observed no difference in the number of pre-synaptic puncta, a result inconsistent with results of previous studies showing the decrease in the number of synapse in the cerebral and cerebellar neurons in hypothyroid animals (62–64). This might be due to the action of TH on synaptogenesis being different in the hippocampus by comparison to the cerebral cortex and cerebellum. As primary cultured neurons form pre- and post-synapses, we also measured mRNA levels of representative proteins involved in synaptic functions (65). Significant decreases in presynaptic protein mRNAs, *Syn1* were observed in -T_3_ group on 10 DIV, whereas no difference was observed in postsynaptic protein (*Dlg4*) mRNA (**Figure 5**). These results indicate that, although TH does not affect synaptogenesis, it may affect synaptic function by regulating the expression of synaptic proteins, particularly at the presynaptic level. Previous study demonstrated that the alteration of synaptic transmission and the reduction in paired-pulse facilitation, which was disrupted by the change in calcium-mediated functions in the pre-synapse, occurred in the rodent hippocampus under perinatal thyroid hormone deficiency (11, 13). It have been reported that the post-synaptic secretion of BDNF was induced in the neuronal activity- and calcium-dependent manner (66). BDNF localizes not only post synaptically but also pre synaptically with a higher pre synaptic levels (67, 68). Therefore, the alternation of synaptic transmission and change in calcium-mediated functions might involve in the decline in the BDNF signaling.

The present study demonstrates that the lack of TH transiently induces abnormal dendrite arborization in the cultured hippocampal neuron. Such aberrant dendrite arborization is rescued by addition of 10 ng/ml BDNF. Our results, along with studies in a rat model of hypothyroidism, indicate that the abnormal dendrite arborization in animals may be, at least in part, caused by the decreased expression of BDNF.

## Data Availability Statement

The raw data supporting the conclusions of this article will be made available by the authors, without undue reservation.

## Ethics Statement

The animal study was reviewed and approved by Animal Care and Experimentation Committee of the Gunma University.

## Author Contributions

HY conducted the whole experiment and prepared the data and manuscript. IA and NK designed complete study, supported the whole experiment, and supervised manuscript. IA and NK integrated complete study and supervised manuscript. IA and NK had responsibility for the whole experiment. SI, TS, WM, and YT supervised the whole experiment and helped to draft the manuscript. All authors contributed to the article and approved the submitted version.

## Funding

This work was supported by the Grant-in-Aid for Scientific Research (B) (Grant 18H03379 to NK) and Grant-in-Aid for Early-Career Scientists (Grant 19K16486 to IA) from the Japan Society for the Promotion of Sciences.

## Conflict of Interest

The authors declare that the research was conducted in the absence of any commercial or financial relationships that could be construed as a potential conflict of interest.
