# Supplementary material for: Absence of Thyroid Hormone Induced Delayed Dendritic Arborization in Mouse Primary Hippocampal Neurons Through Insufficient Expression of Brain-Derived Neurotrophic Factor
Source: Front Endocrinol (Lausanne). 2021 Feb 23;12:629100. doi: 10.3389/fendo.2021.629100 (PMC7940752; doi:10.3389/fendo.2021.629100)
Supplement: Supplementary file 1 [file DataSheet_1.docx]

Supplemental figures


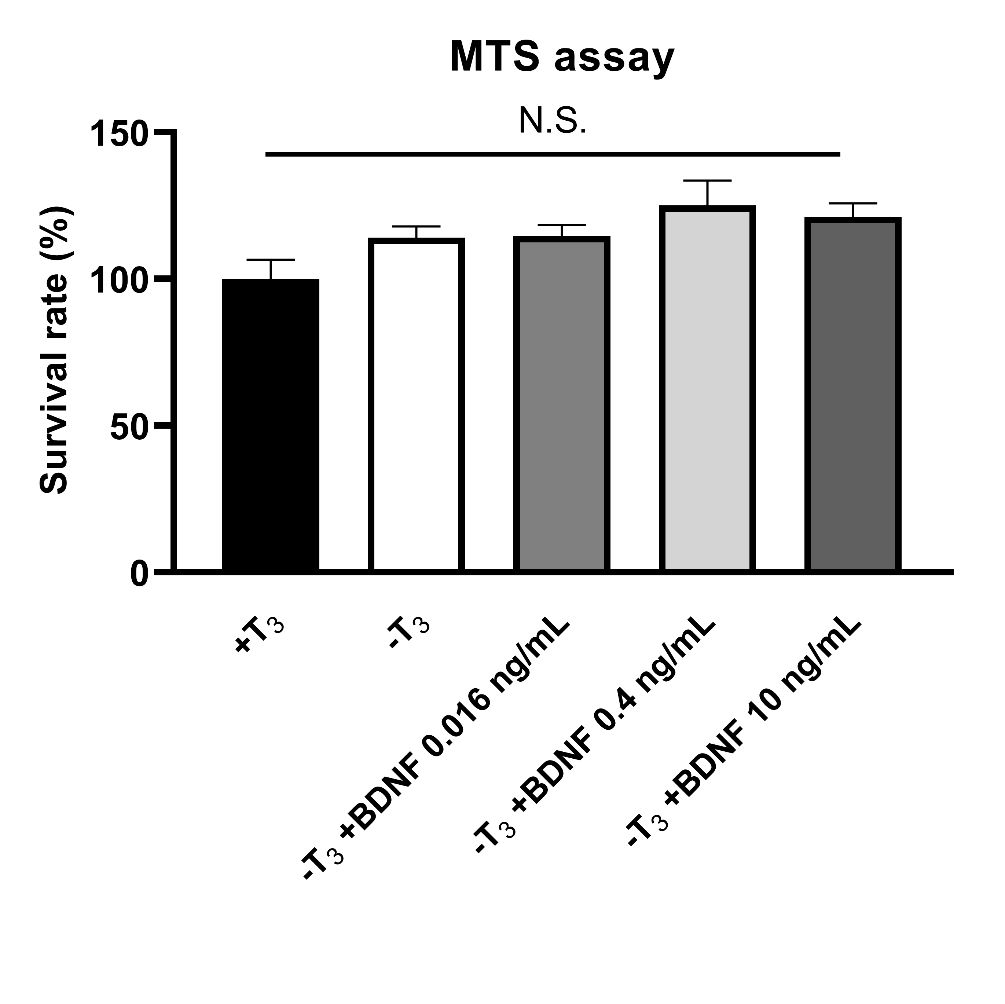


Supplemental figure 1

Absence of T_3_ did not affect cell viability. Cell viability was assessed by MTS assay in each group on 10 DIV. There was no difference among group. Data were obtained from three different dissections (+T_3_ group, n = 3; -T_3_ group, n = 3; -T_3_ +BDNF 0.016 ng/mL group, n = 3; -T_3_ +BDNF 0.4 ng/mL group, n = 3; -T_3_ +BDNF 10 ng/mL group, n = 3). Graph shows mean ± SEM.
